# Climatic Signals in Tree Rings of *Heritiera fomes* Buch.-Ham. in the Sundarbans, Bangladesh

**DOI:** 10.1371/journal.pone.0149788

**Published:** 2016-02-29

**Authors:** Md. Qumruzzaman Chowdhury, Maaike De Ridder, Hans Beeckman

**Affiliations:** 1 Wood Biology Service, Royal Museum for Central Africa (RMCA), Leuvensesteenweg 13, 3080, Tervuren, Belgium; 2 Department of Forestry and Environmental Science, Shahjalal University of Science and Technology, Sylhet, 3114, Bangladesh; Agharkar Research Institute, INDIA

## Abstract

Mangroves occur along the coastlines throughout the tropics and sub-tropics, supporting a wide variety of resources and services. In order to understand the responses of future climate change on this ecosystem, we need to know how mangrove species have responded to climate changes in the recent past. This study aims at exploring the climatic influences on the radial growth of *Heritiera fomes* from a local to global scale. A total of 40 stem discs were collected at breast height position from two different zones with contrasting salinity in the Sundarbans, Bangladesh. All specimens showed distinct tree rings and most of the trees (70%) could be visually and statistically crossdated. Successful crossdating enabled the development of two zone-specific chronologies. The mean radial increment was significantly higher at low salinity (eastern) zone compared to higher salinity (western) zone. The two zone-specific chronologies synchronized significantly, allowing for the construction of a regional chronology. The annual and monsoon precipitation mainly influence the tree growth of *H*. *fomes*. The growth response to local precipitation is similar in both zones except June and November in the western zone, while the significant influence is lacking. The large-scale climatic drivers such as sea surface temperature (SST) of equatorial Pacific and Indian Ocean as well as the El Niño-Southern Oscillation (ENSO) revealed no teleconnection with tree growth. The tree rings of this species are thus an indicator for monsoon precipitation variations in Bangladesh. The wider distribution of this species from the South to South East Asian coast presents an outstanding opportunity for developing a large-scale tree-ring network of mangroves.

## Introduction

Mangroves are carbon rich ecosystems that lie at the interface between land and sea in the tropical and subtropical regions, providing a wide array of resources and services, such as protection against natural calamities, habitats for wildlife and fisheries, support socioeconomic activities and maintain ecological balance [1–5]. Sundarbans, the largest single tract mangrove forest in the world, is situated along the coast of the Bay of Bengal in the estuary of the Ganges–Brahmaputra river, and covers partly Bangladesh (59% of the forest, 6014 km^2^) and India [6]. The Bangladesh Sundarbans is one of the most diverse forests harboring more than 330 plant species, 400 species of fishes, 35 species of reptiles, over 300 species of birds, and 42 species of mammals including the Royal Bengal tiger [7, 8]. Therefore, it has been declared a Ramsar site. Moreover, part of the forest (23%) has been registered as a world heritage site [9]. Despite its enormous importance, the forest is increasingly threatened by excessive livelihood activities, land use changes [9], illegal and overexploitation of forest resources [10], diseases [11], biological invasion [12], natural calamities [13] and oil spills from vessels [14]. In addition, climate change is another important threat to the ecosystem [15, 16].

One of the best-known global climate fluctuations is the El Niño-Southern Oscillation (ENSO) which normally causes anomalous dry and warm conditions over monsoonal Asia during El Niño events [17]. Inter-annual variation in local climate in this region is modulated by large-scale climatic drivers such as sea surface temperature (SST) of the Pacific and Indian Ocean [18]. However, these large-scale climatic phenomena have been shown to affect local climate as well as tree growth over very long distances [19–24]. Therefore, understanding the teleconnections among them is crucial for the prediction of future growth responses to global climatic variation.

Dendrochronology is an important tool for understanding growth dynamics of trees and past environmental changes in the temperate to boreal forest ecosystems [25, 26], but its application is still limited in the tropics due to lack of clear seasonality [25, 27, 28]. Compared to other ecosystems, mangrove species are less studied due to assumption on the absence of clear tree-ring boundaries linked with the highly dynamic intertidal environment [29]. A few earlier studies reported the absence of tree rings [30, 31], or fairly distinct rings (rings present but margins were not necessarily distinct) in some species [32–34]. However, recent studies recurrently reported annual periodicity of growth rings in few mangrove species, such as *Rhizophora*, *Heritiera*, *Sonneratia* and *Laguncularia* spp. [29, 35–39]. Despite distinct tree-ring boundaries in few mangroves species, their dendrochronological application has not been much explored due to crossdating problems [35, 36]. Thus the assessment of dendrochronological potential of a wider range of mangrove species is essential to unravel questions regarding the complexity of the mangrove ecosystem. Recently, the periodicity of tree rings in *Heritiera fomes* Buch.-Ham. has been proved in the Sundarbans using a cambial marking experiment and cambial activity analysis [40]. Moreover, the tree rings of this species synchronized with annual precipitation in Bangladesh [38]. Calibrating tree derived temporal data with climate over the past few decades allows investigation of the complex climate-growth phenomenon and offers the best possible option to understand the response of this ecosystem to future climatic variations [41–43]. Despite such stimulating perspectives, scarcity of adequate proxies from the mangroves largely hinders our efforts to move forward.

*H*. *fomes* is a dominant species in the Sundarbans [44], although its global distribution (from South to South East Asia) has been declining and the species has been categorized as an endangered species [45]. Considering the global concerns and high conservation importance of this species, a better understanding of the growth responses to climate conditions is essential, especially with respect to on-going climate change [46]. This study aims at exploring the dendroclimatological potential of *H*. *fomes* in the Sundarbans, and therefore we address the following questions: i) do the ring-width series crossdate?, ii) which climatic factor mainly influences radial growth? and iii) do the large-scale climatic drivers, such as SST in the equatorial Pacific and Indian Ocean and ENSO influence local climate and/or tree growth?

## Materials and Methods

### Study area and climate

**Fig 1:**
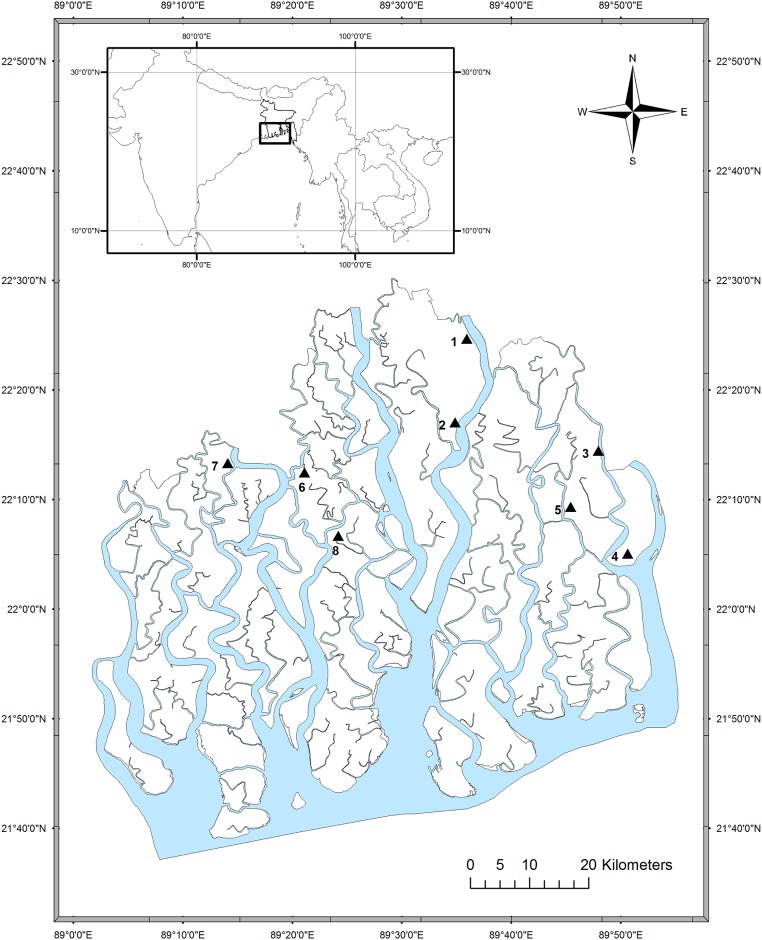
Map showing the sampling locations (triangles) in the Sundarbans. Locations 1 to 5 represent low salinity (eastern), and 6 to 8 are high salinity (western) zone. The map was created using the ArcGIS software (version 10.3, URL: http://www.esri.com/software/arcgis/new).

The study was conducted at Sundarbans which is located in the south-western part of Bangladesh (Fig 1). The forest is characterized by a complex network of branching and meandering distributaries and rivers with a width that varies from a few meters to few kilometers. The main rivers are connected to the Ganges river through the Gorai river and they enclose a collection of low-lying, shifting islands. The tidal amplitude throughout the forests is 3–4 m [47]. Moreover, based on tidal amplitude, the forest can be divided into four zones: inundated by all tides (newly accretions), inundated by normal high tides (covers most of the area), inundated only by spring high tides (mostly in the northern part), and inundated by monsoon high tides (north-eastern part) [9]. The drainage pattern shifts eastward along with the west-to-east tilt, resulting in a substantial reduction in fresh water flow into the western zone, and a natural east (low) to west (high) [9, 47] and north (low) to south (high) direction salinity gradient within the forests [14]. Freshwater discharge within the forest decreases from November to May [48], resulting in an increase of salinity from February to May [14]. Average tree height within the forest also varies from the east (10–20 m) to west zone (<10 m) [9].

**Fig 2:**
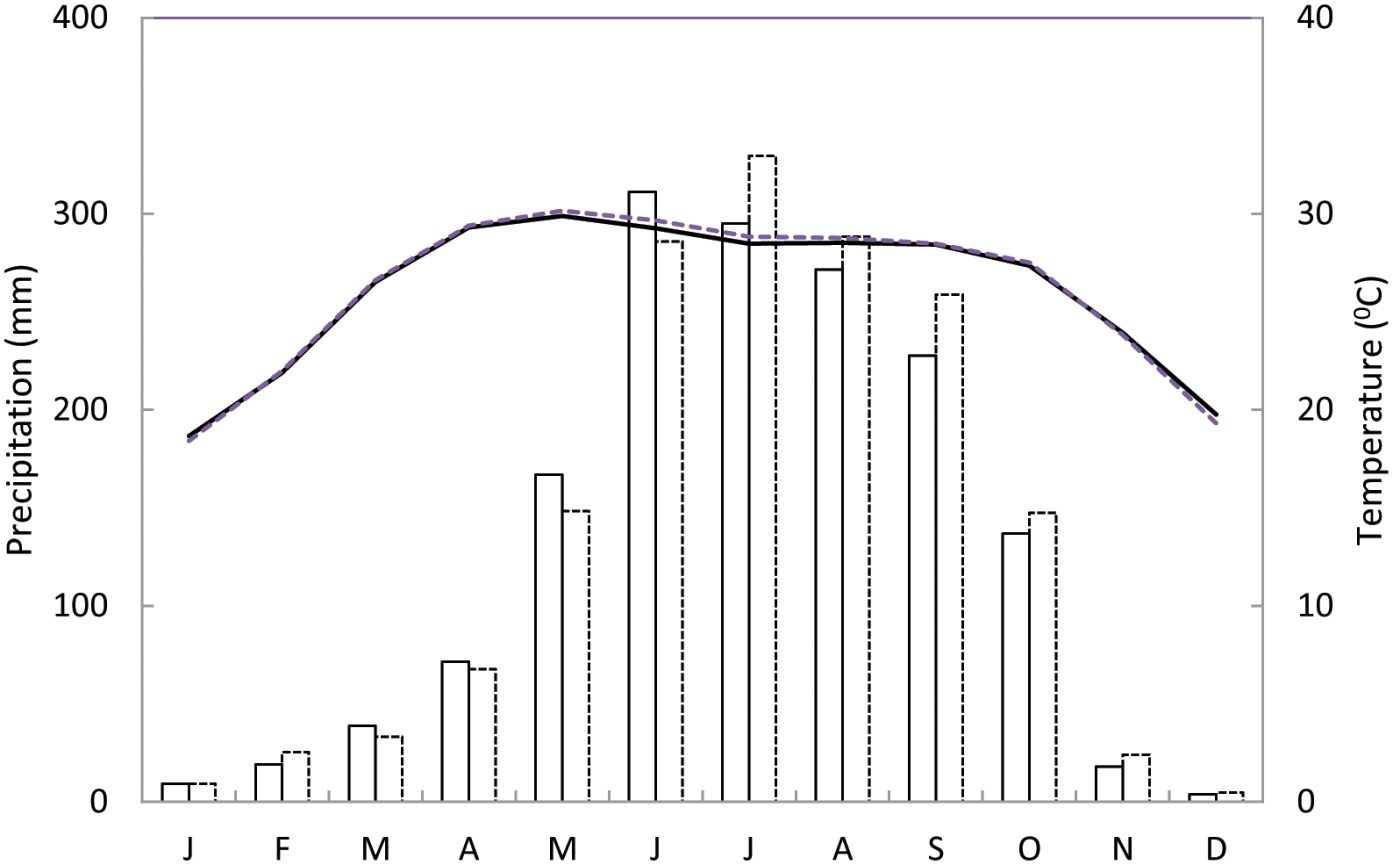
The climate diagram of the study area. The bar and line represent precipitation and temperature, respectively. The solid line is for Khulna station (eastern zone of the study area) and dotted line for Satkhira station (western zone of the study area). Average data for the period of 1948 to 2011.

The study areas are characterized by a monsoonal climate with a unimodal distribution of precipitation (Fig 2). The monsoon ranges from June to September which is preceded by a hot and muggy pre-monsoon (March–May) with sporadic rainfall. The monsoon is followed by a post-monsoon (October–November), and a dry winter (December–February). The climate data is available since 1948 in both stations (Khulna and Satkhira). Distance between both stations is approximately 50 km and climate condition is similar due to close association between precipitation (*r* = 0.48, *p*<0.01) and temperature (*r* = 0.23, *p*<0.05) of both stations. The average temperature of the two stations ranges from 18 to 22°C during winter, and from 27 to 31°C rest of the year. The average relative humidity throughout the year ranges from 69 to 83%. The average annual precipitation is 1600 mm.

### Site variable analysis

Twenty soil samples were collected (during January) in polythene bags from each sampling site (Fig 1) over a depth of 15 cm and brought to the laboratory. The sand, slit and clay percentages were measured using hydrometer method [49]. The electrical conductivity (EC) was determined in a solution of 1:5 soil-water mixtures using a conductivity meter (Extech 341350A-P Oyster) and EC was converted to salinity (ECe) [50]. Salinity variation among the sampling sites was analyzed separately for the eastern zone (site 1, 2, 3, 4 and 5) and the western zone (site 6, 7 and 8) using ANOVA test. In addition, variation between the zones was also analyzed using *t*- test. The inundation classes were denoted as I, II, III and IV as inundated by 100–76%, 75–51%, 50–26%, 25–5% of the high tides, respectively [51].

### Stem disc collection and preparation

A total of 40 stem discs of *H*. *fomes* were collected at breast height (1.3 m above from the ground) position from eight different sites in the Sundarbans (Fig 1). Because of the complex anatomy (for example, frequent occurrence of wedging and partially missing rings) of this species [40], we used stem disc instead of increment core. All stem discs were stored in the Tervuren xylarium, Belgium (accession numbers Tw64638–Tw64677). The stem discs were frozen for one week to prevent insect infestation. All stem discs were sanded using a sanding machine with gradually increasing grit from 150 to 1200.

### Tree-ring analysis

**Fig 3:**
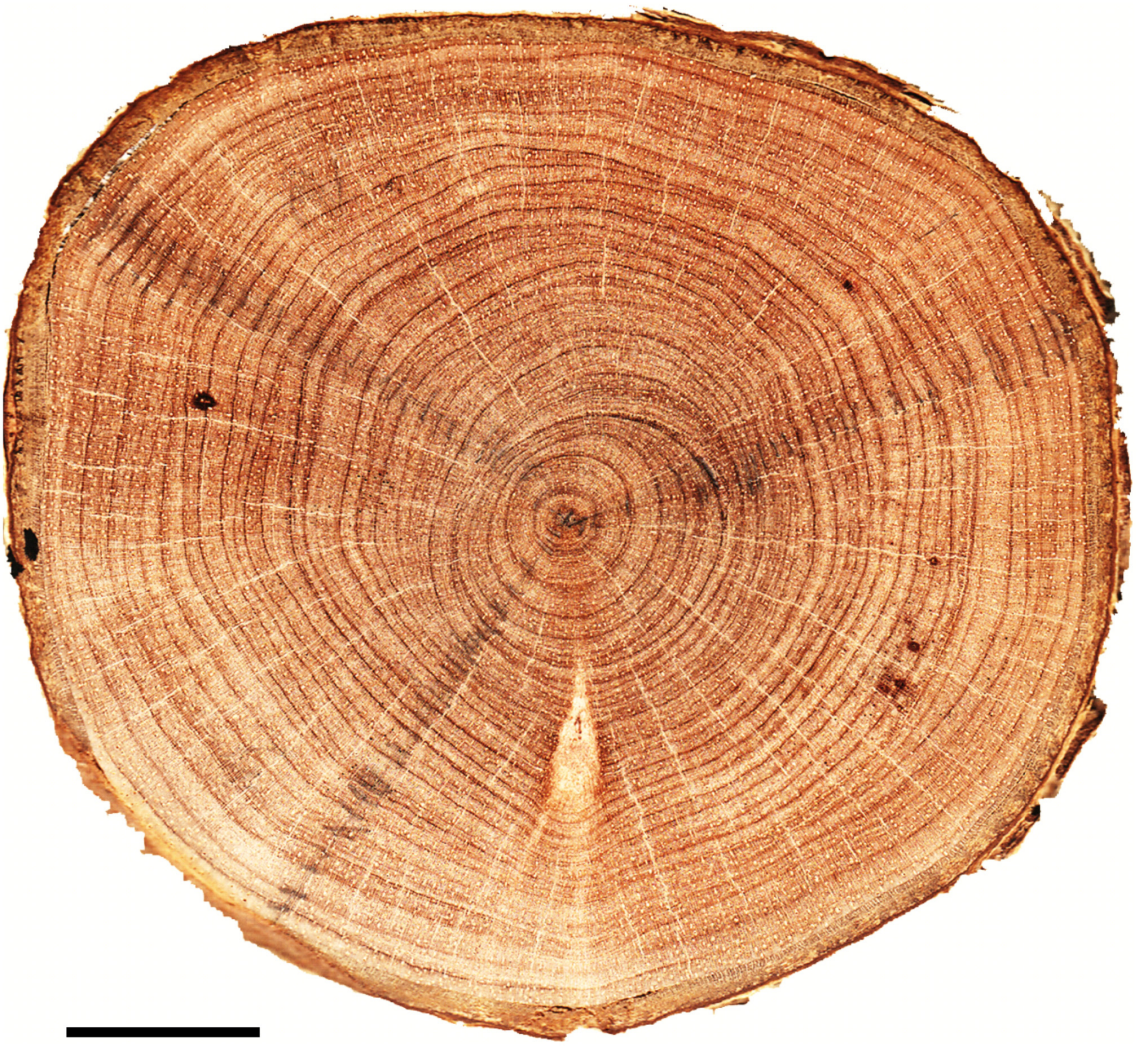
Transversal view of a sanded stem disc showing tree-ring boundaries with pencil marks. Scale bar = 10 mm.

On the sanded discs, tree-ring boundaries were marked with pencil under a stereomicroscope on three radii from pith to bark to check for tree-ring anomalies (Fig 3). Anomalies like wedging and particularly partially missing rings were detected (S1 Fig), and correctly dated the rings in which they formed. Crossdating was performed among the radii of each stem disc by direct comparison of the stem disk and composite skeleton plot was drawn for each tree. If successful, crossdating between trees was done by aligning tree composite skeleton plots [52]. Same procedure was subsequently followed for each site and zone. All pencil marked stem discs were scanned at 2400 dpi and the ring widths were measured using Fiji ImageJ software [53]. Spaghetti plots for all radii were also drawn for comparison (S2 Fig). Correspondence between different trees was checked to ensure that each individual tree ring is assigned to the exact calendar year [54]. In addition, quality of crossdating was verified by considering statistical parameters such as Student’s *t-* [55], *Gleichläufigkeit* values (*GLK*) [56] using TSAP-Win software [57]. The crossdating threshold was set at a *t*- value of 2.0 (*p*<0.05) and a *GLK* of 60% [58, 59]. These thresholds are lower than the thresholds (*t*>3.5 and *GLK*>70%) used in regular dendrochronological practices in temperate and boreal regions where the reference chronologies are available. However, there is no reference chronology available for mangroves or other terrestrial species of nearby area of our study site and therefore individual tree-ring series were compared to other individual tree-ring series rather than to a chronology. In addition, the harvesting date is known and dating of the problematic (wedging or partially missing) rings is possibly an approximation, and the range of possible values is relatively narrow [58]. The thresholds were thus lowered for dating the floating time series because of a narrow range of possible dates is likely [19].

Tree-ring indices were calculated by dividing each of the original tree-ring widths by the value of the fitted spline using the ARSTAN program [60], and developed zonal and regional chronologies. Each growth year was repeated at least four trees to be part of the chronology. Auto-correlation (AC) was calculated to assess the influence of the previous year’s growth upon the current year’s growth [61]. The expressed population signal (EPS) assesses the degree to which the chronology represents a hypothetical chronology based on an infinite number of samples; an EPS of 0.85 is usually taken to identify the reliable part of a tree-ring chronology [62]. The mean sensitivity (MS) is a measure of the mean relative changes between adjacent ring widths and was calculated for the standardized chronologies [61]. In addition, the Pearson’s correlation (*r*) was calculated.

### Climate-growth analysis

The influence of local climate (precipitation, temperature and relative humidity) on tree growth was analyzed using DendroClim 2002 [63] over the common time span from 1948 to 2011. In addition to the individual month, we used the different time periods encompassing annual, pre-monsoon, monsoon and post-monsoon precipitation. The precipitation of these time periods of the previous year was also used in the analysis. Zonal chronologies were correlated with climate data of the nearby meteorological stations: the chronology of the eastern zone with the Khulna and the western zone with the Satkhira station. The regional tree-ring chronology, including both eastern and western zone, was correlated with the average climate data of both climate stations.

Next, we analyzed the relation between large-scale climatic drivers (SST of the equatorial Pacific and Indian Ocean) and local climatic variables (*e*.*g*., precipitation) and their direct influences on tree growth. Spatial correlation maps were generated using the KNMI explorer [64, 65] and were based on gridded (1x1°) monthly and seasonal SST fields (Hadley Centre HadSST3) [66, 67]. Correlations were calculated over the same time span of instrumental climate data (1948 to 2011), and spatial correlation maps were generated to indicate which oceanic regions influence precipitation patterns and tree growth (*p*<0.10). In this study, we used the SST indices for the Niño3.4 region, as this is the traditional region used to assess the strength of El Niño events [68]. We also generated spatial correlation maps (*p*<0.10) between the annual Niño 3.4 index and gridded (1x1°) seasonal precipitation fields (CRU T.S3.0) [69]. We tested the influence of El Niño and La Niña events on tree growth using *t*- test for differences between El Niño/La Niña [70, 71] and ‘normal’ years. For ‘normal’ years, we used the corresponding next year of each event [24]. Finally, we explored the correlation of the regional chronology and monsoon precipitation with the Indian summer monsoon index [72].

## Results

### Site variation

The salinity variation was not significant among the sampling sites either in the eastern (F_4,15_ = 2.75, *p*>0.05) or western zone (F_2,17_ = 2.90, *p*>0.05). However, salinity variation between the eastern and western zone was significant (*t* = 8.42, df = 38, *p*<0.01), and the western zone showed higher salinity (Table 1). The inundation category did not show clear relationship with salinity variation but sites with higher inundation category (category I) showed lower salinity.

**Table 1:** Summary of the site characteristics and radial increment of the sampled trees.

Zone	Sampling site	^a^Dbh±^b^stdev (cm)	Salinity±^b^stdev (ECe; dS m^-1^)	Inundation category*	^c^RW±^b^stdev (mm)
**East**	1	19.5±4.3	20±2	II	1.20±0.18
	2	13.5±2.7	21±3	II	1.08±0.15
	3	7.6±0.9	18±4	I	1.19±0.24
	4	9.8±2.6	19±4	II	1.14±0.16
	5	20.6±5.5	16±5	I	1.24±0.12
**West**	6	9.5±0.7	37±5	II	0.95±0.10
	7	13.4±1.8	35±7	III	0.93±0.22
	8	18±1.6	42±3	III	0.90±0.15

^a^Dbh, diameter at breast height;

^b^stdev, standard deviation;

^c^ RW, ring width;

*, Inundation category according to Tomlinson [51]

### Tree-ring measurements and chronology building

Concentric tree rings are microscopically visible in all samples (Fig 3), and the tree-ring boundaries are marked by a marginal parenchyma band, predominantly one cell wide but occasionally up to three cells and occasionally mixed with fibers [38, 40]. The wedging and partially missing rings were found in the samples of the eastern and western zone (S1 Fig). These problematic rings were detected and dated correctly by checking the whole stem disc. The ring width significantly varied between the zones (*p*<0.05) and the eastern zone showed higher value (1.17 ± 0.16 mm yr^-1^) than the western zone (0.93 ± 0.15 mm yr^-1^). However, ring-width variation within the zone was not significant in both cases. The overall mean tree-ring width was 1.08 ± 0.16 mm yr^-1^ (Table 2).

**Table 2:** Characteristics of the eastern, western and regional chronologies.

Variables	Eastern Zone	Western zone	Regional
**Total no. of samples**	27	13	40
**Mean Dbh ± stdev (cm)**	14.4±6.2	13.7±4.2	14.2±5.6
**Mean age**	55	61	57
**Mean growth rate ± stdev (mm)**	1.17±0.16	0.93±0.15	1.08±0.16
**No. of trees in chronology (% total no. of trees)**	20 (74%)	8 (62%)	28 (70%)
**Time span of chronology**	72 (1940–2011)	68 (1944–2011)	77 (1935–2011)
^a^**GLK (%)**	68	71	68
^b^**TVBP**	2.2	2.6	2.4
^c^**EPS**	0.90	0.91	0.90
^d^**AC (before standardization)**	0.22	0.20	0.22
^e^**MS**	0.35	0.34	0.35
**Pearson correlation (*r*)**	0.33	0.36	0.35

^a^*GLK* (Gleichläufigkeit);

^b^TVBP, *t*- value of Baillie and Pilcher [55];

^c^EPS (expressed population signal);

^d^AC (auto-correlation);

^e^MS (mean sensitivity)

**Fig 4:**
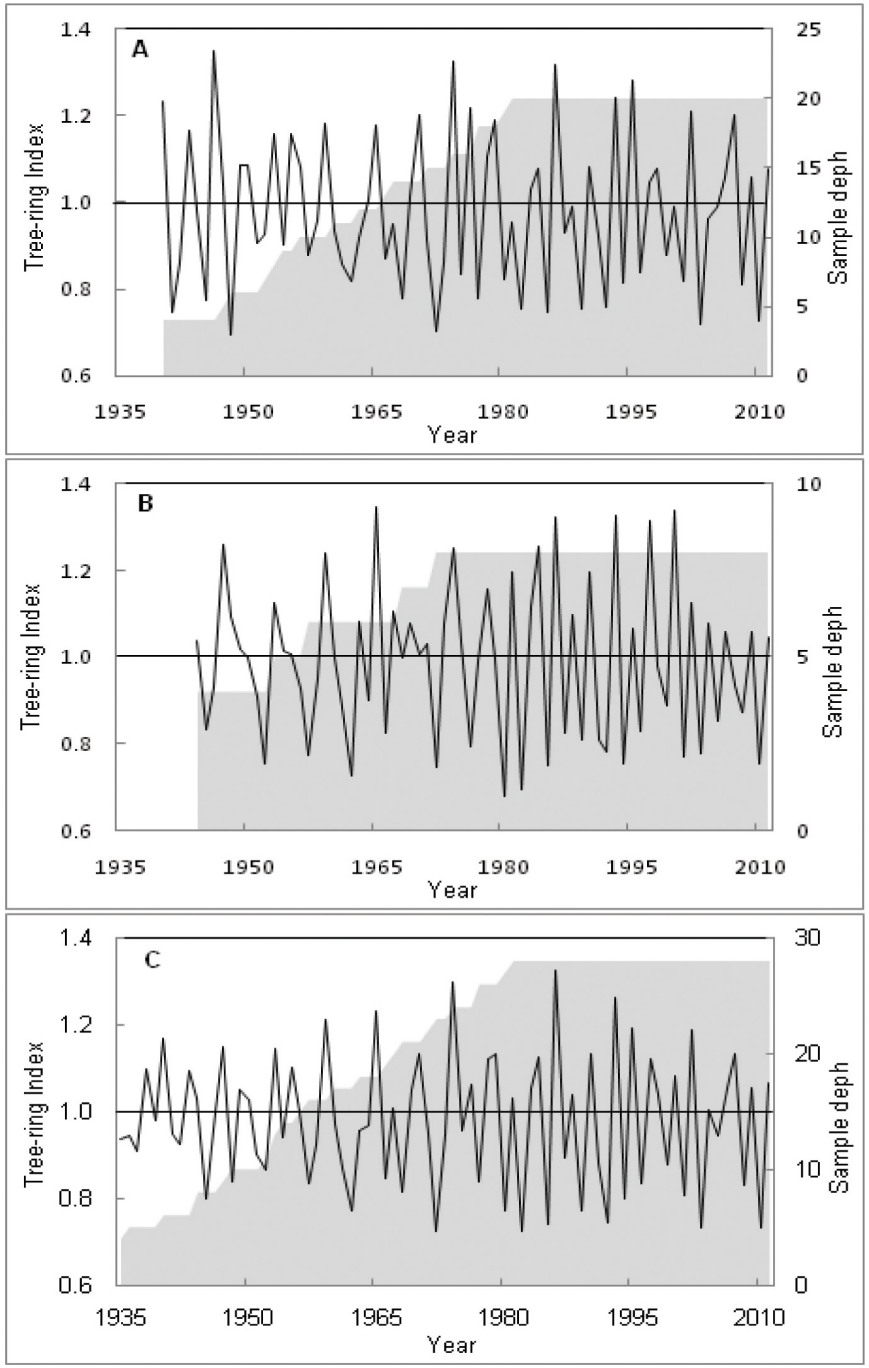
The standardized chronologies of the eastern (A) and western (B) zone. The regional chronology is shown in C. Solid line indicates the chronology and shadow indicates the sample depth in each case.

Despite frequent tree-ring anomalies (wedging and partially missing rings), crossdating between tree-ring series of 28 trees was successful (Table 2). Two zone-specific tree-ring chronologies were developed (Fig 4A and 4B; Table 2) covering respectively 72 (1940–2011) and 68 (1944–2011) year in the eastern and western zone (Table 2). The pattern on both zonal chronologies was similar up to 2000 and then the chronology of western zone showed a decreasing trend (Fig 4B). Based on the crossdating criteria and significant correlation (*r* = 0.60, *p*<0.01) between two zonal chronologies, we therefore developed a regional chronology covering 77 (1935–2011) year (Fig 4C; Table 2). The EPS of the east, west and regional chronologies was equal or higher than 0.90 and shows that our samples capture around 90% of the inter-annual growth variation in the population. The auto-correlation (AC) of the three chronologies was low (Table 2). On the other hand, the mean sensitivity (MS) and the Pearson correlation of three chronologies were similar and showed moderate values (Table 2).

### Climate-growth relationships

**Fig 5:**
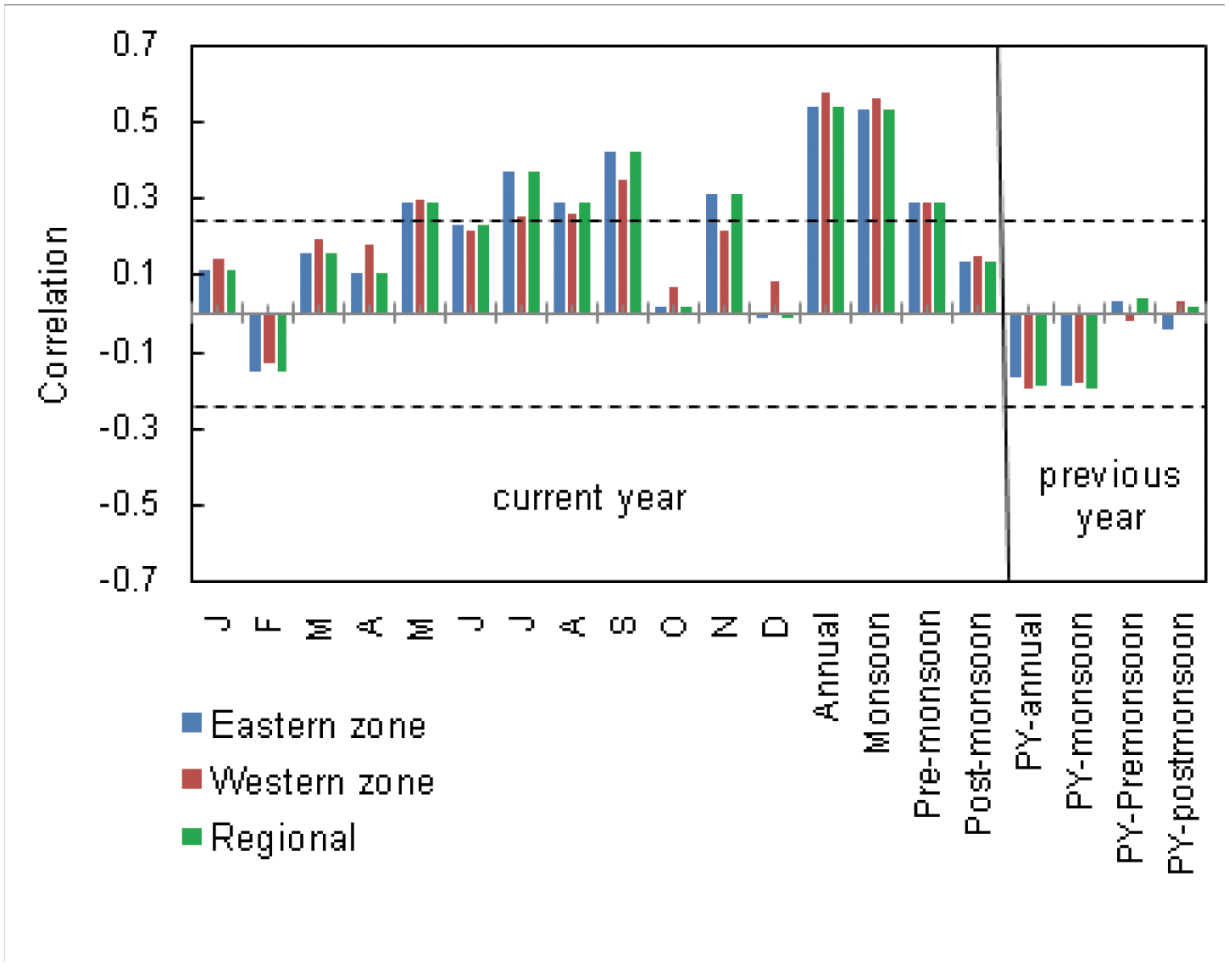
Correlation coefficients between the tree-ring chronologies and precipitation data (1948–2011). The chronology of eastern and western zone was correlated with precipitation of Khulna and Shakhira district, respectively. The regional chronology was correlated with regional (average of both districts) precipitation. For current year, the monthly, annual and seasonal, such as monsoon (June–September), pre-monsoon (March–May) and post-monsoon (October–November) precipitation was used for analysis. In case of previous year (PY-), the annual, monsoon, pre-monsoon and post-monsoon precipitation was used. Dotted horizontal lines indicate 95% significance level and solid vertical line is the boundary between current and previous year.

Increased precipitation during May to September and November enhanced local tree growth in the eastern zone (Fig 5). Trees in the western zone showed similar relationships, except the precipitation of June and November. On the regional scale, precipitation of May to September and November showed positive correlation (Fig 5). The annual precipitation yielded the highest correlations with the three chronologies (east, *r* = 0.52; west, *r* = 0.57; regional, *r* = 0.54) followed by monsoon (east *r* = 0.51; west *r* = 0.55; regional *r* = 0.53) and pre-monsoon (east, *r* = 0.25; west, *r* = 0.29; regional, *r* = 0.29) precipitation (Fig 5). Post-monsoon precipitation was not significantly correlated with the chronologies. Similarly, the precipitation of previous year did not show significant correlation with any of the chronologies. In addition, the monthly temperature and relative humidity did not show significant correlations with chronologies.

**Fig 6:**
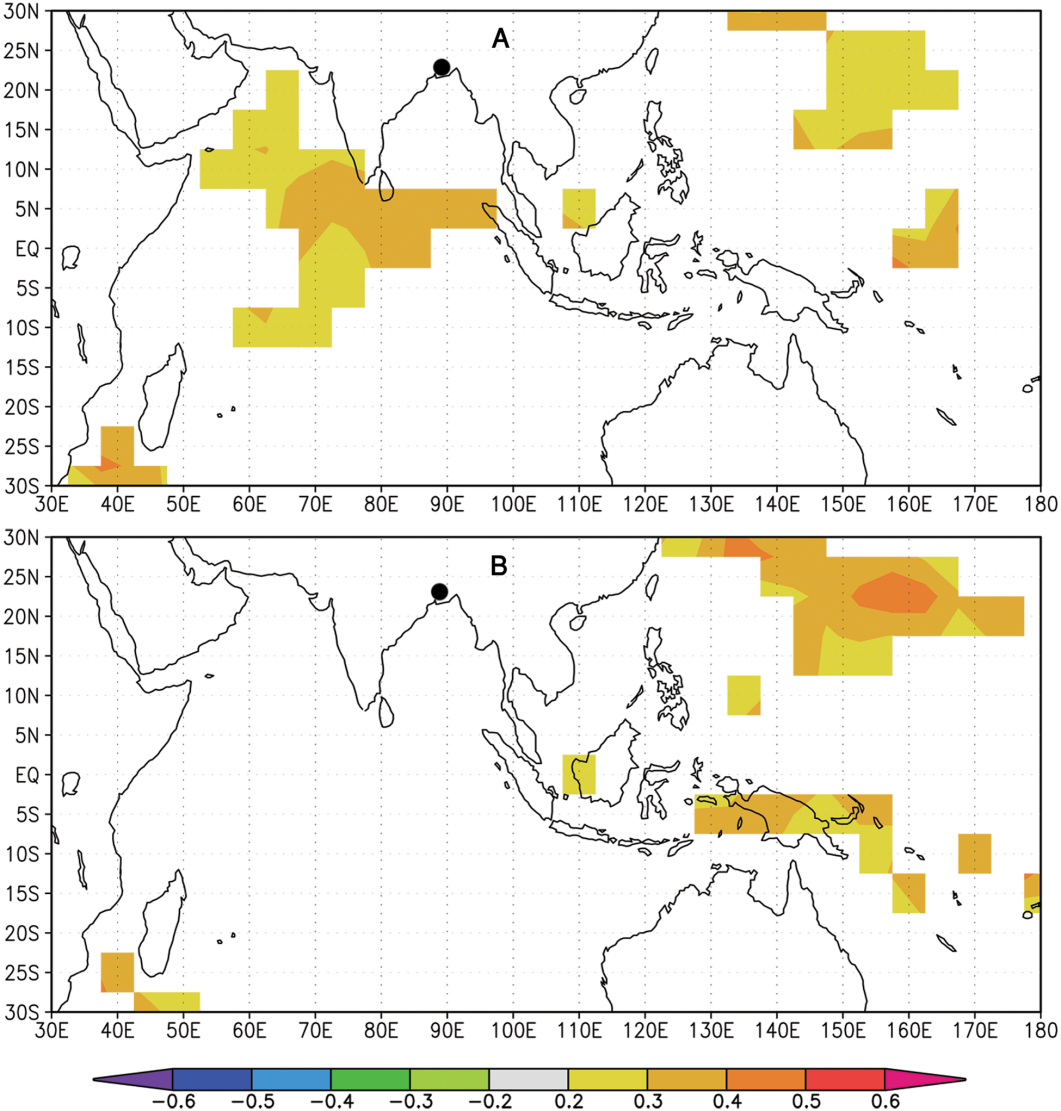
The correlation (1948–2011) between the gridded sea surface temperature (SST; Hadley Centre HadSST3) and precipitation during August–November (A), and September–December (B). Black circle indicates the study area. Orange and red colors indicate positive correlations, and green and blue colors indicate negative correlations (*p*<0.10). The correlation map was created using the KNMI Climate Explorer (The Royal Netherlands Meteorological Institute; http://climexp.knmi.nl/).

**Fig 7:**
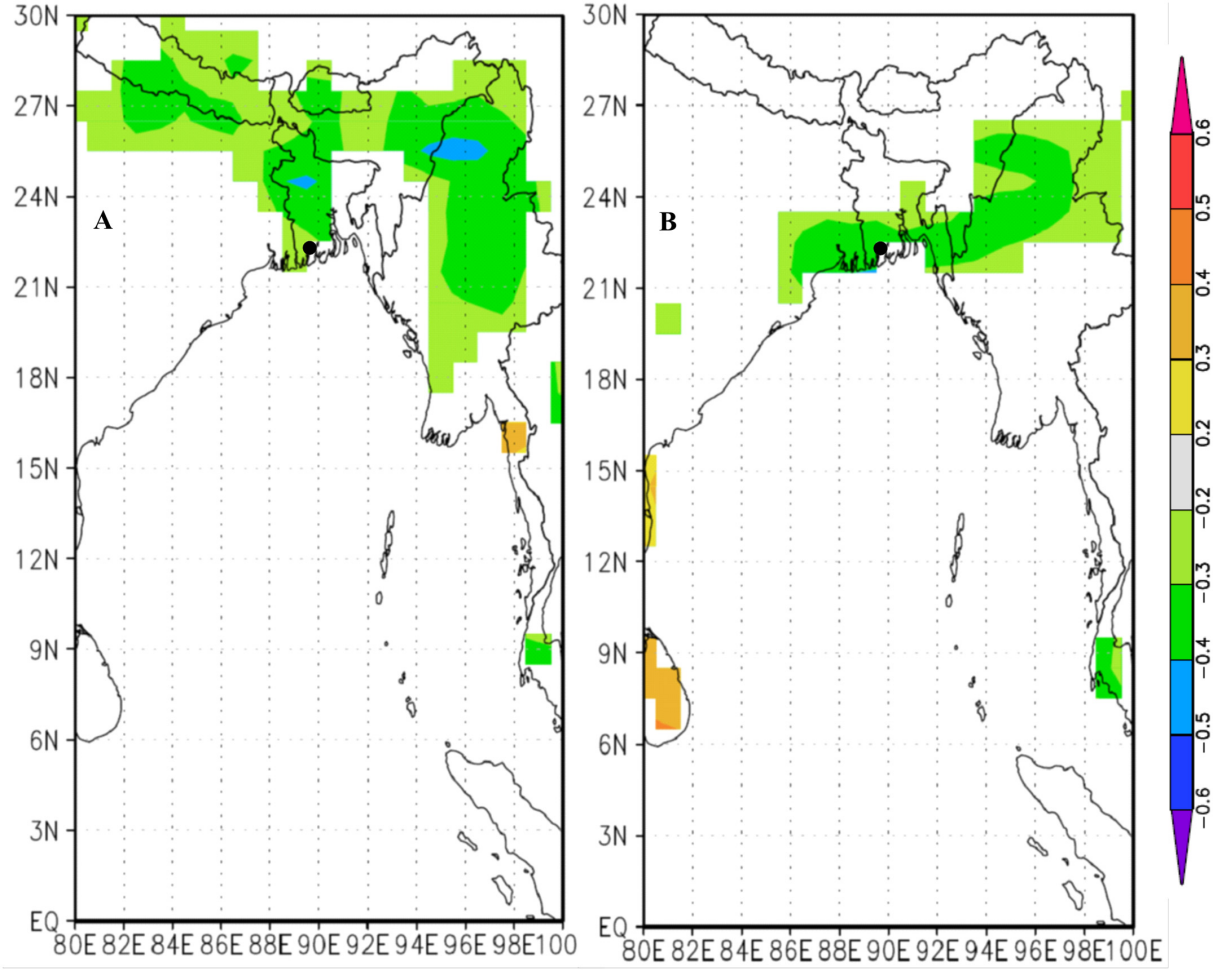
The correlation (1948–2011) between the El Niño 3.4 time-series (gridded 1x1°) and gridded May–July (A), and October–December (B) precipitation (CRU T.S3.0; gridded 1x1°). Black circle indicates the study area. Orange and red colors indicate positive correlations, and green and blue colors indicate negative correlations (*p*<0.10). The correlation map was created using the KNMI Climate Explorer (The Royal Netherlands Meteorological Institute; http://climexp.knmi.nl/).

We found significant correlations for regional precipitation with gridded sea surface temperature (SST) for the Pacific from September to December and Indian Ocean from August to November (Fig 6). The SST of both oceans during this time period or other periods of the year was not directly correlated with the regional tree-ring chronology. At a global scale, precipitation of May to July and October to December was negatively influenced by ENSO (Fig 7). However, tree growth was not influenced by this force, even in the aforementioned time periods. Moreover, tree growth in strong El Niño and La Niña years did not differ significantly from the corresponding ‘normal’ years. The Indian summer monsoon index [72] showed poor correlation with local precipitation (*r* = -0.12, n = 64) and radial growth (*r* = -0.03, n = 64).

## Discussion

### Tree-ring characteristics and chronology development

Despite the distinct character of boundaries (Fig 3), tree-ring anomalies, mostly wedging rings might be associated with phases of low growth rates or eccentric growth occurring in this species [40], and also in other tropical species [58, 59,73]. Approximately, 70% trees from the both sites could be visually and statistically crossdated which confirms the annual nature of tree rings. This result is consistent with the previous study [40], where the annual nature of tree ring of this species was tested by integrating a cambial marking experiment and cambium activity analysis. The aforementioned study shows that cambial dormancy occurs in *H*. *fomes* from January to April in the Sundarbans. Because there was no new restored wood and the growth ring boundary began immediately after the cambial marking (in January) and presence of dormant cambium in the samples harvested in March to April. It is noted that a cambial dormancy lasting more than three months is sufficient to trigger the ring formation in tropical species [74, 75]. In addition, synchronization with annual precipitation is also an indicator for existence of annual tree ring in this species [38].

In the eastern zone, the mean radial increment of *H*. *fomes* is considerably higher than that of the western zone (Table 2). We might speculate that such inter-zone growth differences resulted mainly from variations in growth condition. Within a homogenous climatic region, the eastern zone is characterized by lower soil salinity than the western zone (Table 1). The higher salinity stress combined with the lower inundation frequency might be reflected as more unfavorable growth conditions [29, 35]. However, the radial increments of this study are similar with the previous study on this species [38, 40]. The growth of this species is also in agreement with the relatively slow growth rate of other mangrove species studied in Kenya showing a range of 0–5 mm yr^-1^ in both *R*. *mucronata* Lam. and *A*. *marina* (Forssk.) Vierh. [36, 76], *Xylocarpus granatum* J. König (0.62–2.51 mm yr^-1^), *Bruguiera gymnorhiza* (L.) Lam. (0–2.51 mm yr^-1^), *R*. *mucronata* (0.94 mm yr^-1^) and *S*.*alba* Sm. (0.31–1.25 mm yr^-1^) from Micronesia [77], *A*. *marina* 1.19 mm yr^-1^ from Australia [78]. We compared the radial growth rate in different continents but it appears precarious due to different confounding factors, such as environment, ontogeny and inter-generic differences, which need to be taken into account to compare growth data [29].

The most important challenges encountered during crossdating were detecting problematic (wedging and partially missing) rings (S1 Fig). However, crossdating success in most of the trees in both zones (Table 2), and strong correlation (*r* = 0.60, *p*<0.01) between both chronologies indicate that there is a common external factor influencing the growth of this species [60, 74], and lead to develop a regional chronology (Fig 4C). The auto-correlation (AC) is considerably low in each case (Table 2), which might be due to stabilizing residuals after averaging individual chronology. Moreover, this is a shade tolerant species [9], which grows slowly during the juvenile stage and thus a strong growth trend is lacking. The mean sensitivity *i*.*e*. year-to-year variability in radial growth is moderate and low values of auto-correlation (Table 2) indicating that the species might response to the annual environmental changes in the Sundarbans.

### Influence of climate

The climate-growth relationship in both zones is similar, except June and November when significant influence is lacking in the higher salinity (western) zone. The annual precipitation positively influenced tree growth from local to regional level (Fig 5), suggesting that fresh water availability is one of the important limiting factor for radial growth. A strong monsoonal impact on radial growth is to be expected as 71% of the annual precipitation occurs during this season in the study area. As predicted, the chronologies exhibit strong correlations with monsoon (June–September, Fig 5) precipitation, representing the main growing season of this species. Earlier studies on subtropical mangrove species in the South Florida also reported that diameter growth in mangrove species certainly linked to precipitation, and trees can produce 75 to 90 percent of their annual radial increments during the wet season [79]. The rate of photosynthesis and stomatal conductance also increased in Kenyan *R*. *mucronata* during the wet season [80], indicating higher growth rate with higher precipitation. For other terrestrial species, like Teak (*Tectona grandis* L.), tree growth in a similar monsoonal climate in Myanmar is positively correlated with precipitation during and prior to the monsoon (May–September) [23, 28].

Precipitation influences soil salinity in mangrove forests [9]. Hence, this is a promising candidate to explain at least a part of the annual growth variations. Not only precipitation but also river discharge, land run-off and tidal inundation [48] that extends up to 50 km inland [81] influence soil water salinity, resulting in considerable spatial and temporal salinity variations in the Sundarbans [14, 82]. Recorded observations also indicate that the up-steam river flow decreases more than 75% from December to April [48] while the water salinity increases around 80–90% within the Sundarbans [14]. The positive correlations between pre-monsoon (March–May) precipitation (Fig 5) and three chronologies suggest that before starting of the new growing season it might diminish the soil salinity and relieve the saline stress to trees. This could be reflected in the timing of onset of the growing season that indicates by flushing new leaves in May to June [40]. Still, there are some positive effects of November precipitation on the eastern and regional chronology (Fig 5), which probably maintains low saline soil- or stem-water content and thereby creating high water potentials *e*.*g*., [83] that might favor tree growth during this season.

Tree-ring data of the Indian sub-continent have been reported to be linked with the SST which could be used for teleconnections among different climate phenomena [20, 21, 23]. The spatial analysis with the SST data shows a distinct pattern of relationship with local precipitation, dominated by a significant positive correlation with the Indian Ocean from August to November and during September to December for the central equatorial Pacific (Fig 6). However, we did not observe significant influences between tree growth and SST of both oceans, even in the aforementioned time periods. The anomalous ENSO conditions are also not associated with tree growth, even though local precipitation has strong influences on that. Precipitation of May to July and October to December was negatively influenced by ENSO (Fig 7), indicating that El Niño/La Niña years resulting in drier/wetter than normal years. However, instrumental data of local precipitation in the ENSO influencing period (May–July; October–December) is not consistent with all El Niño or La Niña years as observed in previous studies [70, 71]. For example, precipitation of the strong El Niño year 1997 is not drier than normal year and similarly La Niña years 1973 and 1975 is not much wetter. Moreover, the correlation patterns also vary between two time periods, such as ENSO from May to July mainly influences the western part whereas during October to December in southern part of the country (Fig 7). The influences of ENSO on precipitation occur in the early and later part of the growing season and could have lesser influence on tree growth. In a similar monsoonal climate (Myanmar), *T*. *grandis* correlates negatively with Niño-3 SST, consistent with the tendency for El Niño events to be linked to drought over Southeast Asia [23]. It is exhibited that ENSO events do have impacts on monsoon systems on sub-continental scale [70, 71] which suggests that local precipitation variability is related to other oceanic patterns, or only to local/regional atmospheric dynamics *e*.*g*., [84]. The Indian summer monsoon index [72] showed poor correlation with local precipitation, an agreement with the earlier studies [68, 85], and so the radial growth of trees. This states that the monsoon precipitation over Bangladesh is generally not correlated with India.

## Conclusions

For the first time, we present the dendroclimatological potential of *H*. *fomes* from Bangladesh mangroves. Its tree rings are well-defined and crossdatable which leads to the development of a regional tree-ring chronology. Despite distinct differences in site conditions such as salinity, both study zones showed similar growth patterns. The tree growth is mainly influenced by the local climate, such as monsoon precipitation. Still there are many high diameter trees (>50 cm) with nearly 200 years life-span [40], which certainly offers the opportunity to construct a longer chronology. The influences of large scale climatic drivers (SST and ENSO) on tree growth were not significant. However, these drivers influenced the local climate only for some periods. Therefore, understanding the climate-growth relationships will be helpful to make inferences about the responses of this ecosystem to future climatic change. Summarized a longer chronology with a good site replication would improve our understanding of the climate/growth association for this species and support the construction of a tree-ring network within the natural range of *H*. *fomes*.

## Supporting Information

S1 Fig Wedging ring on transverse section of a sanded stem disc (arrow indicated). Scale bar = 5 mm.
(TIF)

S2 Fig The spaghetti plots of cross dated tree-ring series of the eastern (A) and western (B) zone. Combined tree-ring series of both zones is shown in C. (TIF)

## References

[1] Kathiresan K, Bingham BL. Biology of mangroves and mangrove ecosystems. Advan Mar Biol. 2001; 40: 81–251. 10.1016/S0065-2881(01)40003-4

[2] Mumby PJ, Edwards AJ, Arias-Gonzalez JE, Lindeman KC, Blackwell PG, Gall A, et al Mangroves enhance the biomass of coral reef fish communities in the Caribbean. Nat. 2004; 427: 533–536. 10.1038/nature02286 14765193

[3] Dahdouh-Guebas F, Jayatissa LP, Di Nitto D, Bosire JO, Lo Seen D, Koedam N. How effective were mangroves as a defence against the recent tsunami? Curr Biol. 2005; 15: 443–447. 10.1016/j.cub.2005.06.008 15964259

[4] Donato DC, Kauffman JB, Murdiyarso D, Kurnianto S, Stidham M, Kanninen M. Mangroves among the most carbon-rich forests in the tropics. Nat Geosci. 2011; 4: 293–297. 10.1038/ngeo1123

[5] Lee SY, Primavera JH, Dahdouh-Guebas F, McKee K, Bosire JO, Cannicci S, et al Ecological role and services of tropical mangrove ecosystems: A reassessment. Global Ecol Biogeogr. 2014; 23: 726–743. 10.1111/geb.12155

[6] Chaudhuri AB, Choudhury AC. Mangroves of the Sundarbans. vol 1 India, Bangkok: IUCN; 1994.

[7] IUCN. The Bangladesh Sundarbans: a photo real sojourn. Dhaka: IUCN-The World Conservation Union Bangladesh country office, Dhaka; 2001.

[8] Islam MS, Wahab MA. A review on the present status and management of mangrove wetland habitat resources in Bangladesh with emphasis on mangrove fisheries and aquaculture. Hydrobiologia. 2005; 542: 165–190. 10.1007/1-4020-4111-X_19

[9] Siddiqi NA. Mangrove forestry in Bangladesh. Chittagong: Institute of Forestry and Environmental Sciences, University of Chittagong; 2001.

[10] Biswas SR, Choudhury JK, Nishat A, Rahman MM. Do invasive plants threaten the Sundarbans mangrove forest of Bangladesh? For Ecol Manage. 2007; 245:1–9. 10.1016/j.foreco.2007.02.011

[11] Rahman MA. Top dying of Sundri (Heritiera fomes) trees in the Sundarbans: extent of damage Proceedings of the national seminar on the Sundarbans, the largest mangrove forest on the earth: A world heritage site. Khulna: Khulna University; 2003.

[12] Biswas SR, Khan MSI, Mallik AU. Invaders’ control on post-disturbance succession in coastal mangroves. J Plant Ecol. 2012; 5: 147–156.

[13] IUCN. Status of the ecological integrity of the Sundarbans. Dhaka: IUCN Bangladesh Country Office, Dhaka; 2003.

[14] Islam SN, Gnauck A. Mangrove wetland ecosystems in Ganges-Brahmaputra delta in Bangladesh. Front Earth Sci China. 2008; 4: 439–448. 10.1007/s11707-008-0049-2

[15] Agrawala S, Ota T, Ahmed AU, Smith J, Aalst MV. Development and climate change in Bangladesh: focus on coastal flooding and the Sundarbans. Organization for Economic Co-operation and Development (OECD), Paris; 2003.

[16] Loucks C, Barber-Meyer S, Hossain MAA, Barlow A, Chowdhury RM. Sea level rise and tigers: predicted impacts to Bangladesh’s Sundarbans mangroves. Clim Chang. 2010; 98: 291–298. 10.1007/s10584-009-9761-5

[17] Ummenhofer CC, D'Arrigo RD, Anchukaitis KJ, Buckley BM, Cook ER. Links between Indo-Pacific climate variability and drought in the monsoon Asia drought atlas. Clim Dyn. 2013; 40: 1319–1334. 10.1007/s00382-012-1458-1

[18] Cash BA, Rodó X, Kinter JL, Yunus M. Disentangling the impact of ENSO and Indian Ocean variability on the regional climate of Bangladesh: Implications for cholera risk. J Clim. 2010; 23: 2817–2831. 10.1175/2009JCLI2512.1

[19] Schöngart J, Junk WJ, Piedade MTF, Ayres JM, Hüttermann A, Worbes M. Teleconnection between tree growth in the Amazonian floodplains and the El Nino-Southern Oscillation effect. Global Change Biol. 2004; 10: 683–692. 10.1111/j.1529-8817.2003.00754.x

[20] Buckley BM, Cook BI, Bhattacharyya A, Dukpa D, Chaudhary V. Global surface temperature signals in pine ring-width chronologies from southern monsoon Asia. Geophys Res Lett. 2005; 32 10.1029/2005GL023745

[21] Bhattacharyya A, Shah SK. Tree-ring studies in India past appraisal, present status and future prospects. IAWA J. 2009; 30: 361–370.

[22] Brienen RJL, Lebrija-Trejos E, Zudeima PA, Martínez-Ramos M. Climate-growth analysis for a Mexican dry forest tree shows strong impact of sea surface temperatures and predicts future growth declines. Glob Chang Biol. 2010; 16: 2001–2012. 10.1111/j.1365-2486.2009.02059.x

[23] D'Arrigo R, Palmer J, Ummenhofer CC, Kyaw NN, Krusic P. Three centuries of Myanmar monsoon climate variability inferred from teak tree rings. Geophys Res Lett. 2011; 38 10.1029/2011GL049927

[24] Ridder De, Trouet V, den Bulcke JV, Hubau W, Acker JV, Beeckman H. A tree-ring based comparison of *Terminalia superba* climate-growth relationships in West and Central Africa. Trees. 2013a; 27: 1225–1238. 10.1007/s00468-013-0871-3

[25] Schweingruber FH. Tree rings—basics and applications of dendrochronology Dordrecht: D. Reidel Publishing Company; 1988.

[26] Schweingruber FH. Tree rings and environment: Dendroecology Swiss Federal Institute for Forest, Snow and Landscape Research: Paul Haupt Verlag, Bern; 1996.

[27] Worbes M. One hundred years of tree-ring research in the tropics—a brief history and an outlook to future challenges. Dendrochronologia. 2002; 20: 217–231. 10.1078/1125-7865-00018

[28] Pumijumnong N. Dendrochronology in Southeast Asia. Trees. 2013; 27:343–358. 10.1007/s00468-012-0775-7

[29] Robert EMR, Schmitz N, Okello JA, Boeren I, Beeckman H, Koedam N. Mangrove growth rings: fact or fiction? Trees. 2011; 25: 49–58. 10.1007/s00468-010-0487-9

[30] Van Vliet GJCM. Wood anatomy of the Rhizophoraceae. Leiden Bot Ser. 1976; 3: 20–75.

[31] Sun Q, Suzuki M. Wood anatomy of mangrove plants in Iriomote Island of Japan: a comparison with mangrove plants from lower latitudes. Acta Phyt Geobot. 2000; 51: 37–55.

[32] Amobi CC. Periodicity of wood formation in twigs of some tropical trees in Nigeria. Ann Bot. 1974; 38: 931–936.

[33] Rao RV, Sharma B, Chauhan L, Dayal R. Reinvestigations of the wood anatomy of Duabanga and Sonneratia with particular reference to their systematic position. IAWA Bull. 1987; 8: 337–345.

[34] Srivastava R, Suzuki M. More fossil woods from the palaeogene of northern Kyushu, Japan. IAWA J. 2001; 22: 85–105.

[35] Menezes M, Berger U, Worbes M. Annual growth rings and long-term growth patterns of mangrove trees from the Bragança peninsula, North Brazil. Wetlands Ecol Manage. 2003; 11: 233–242.

[36] Verheyden A, Kairo JG, Beeckman H, Koedam N. Growth rings, growth ring formation and age determination in the mangrove *Rhizophora mucronata*. Ann Bot. 2004; 94: 59–66. 10.1093/aob/mch115 15145790 PMC4242371

[37] Yu KF, Kamber BS, Lawrence MG, Greig A, Zhao JX. High precision analysis on annual variations of heavy metals, lead isotopes and rare earth elements in mangrove tree rings by inductively coupled plasma mass spectrometry. Nucl Instrum Method. 2007; 255: 399–408. 10.1016/j.nimb.2006.11.127

[38] Chowdhury MQ, Schmitz N, Verheyden A, Sass-Klaassen U, Koedam N, Beeckman H. Nature and periodicity of growth rings in two Bangladeshi mangrove species. IAWA J. 2008; 29: 265–276.

[39] Estrada GCD, Callado CH, Soares MLG, Lisi CS. Annual growth rings in the mangrove Laguncularia racemosa (Combretaceae). Trees. 2008; 22: 663–670. 10.1007/s00468-008-0224-9

[40] Chowdhury MQ, Kitin P, De Ridder M, Delvaux C, Beeckman H. Cambial dormancy induced growth rings in Heritiera fomes Buch.-Ham.: a proxy for exploring the dynamics of Sundarbans, Bangladesh. Trees. 2015; in press. 10.1007/s00468-015-1292-2

[41] Briffa KR, Schweingruber FH, Jones PD, Osborn TJ, Harris IC, Shiyatov SG, et al Trees tell of past climates: but are they speaking less clearly today? Philos Trans R Soc London Ser B—Biol Sci. 1998; 353: 65–73. 10.1098/rstb.1998.0191

[42] Verheyden A, Ridder FD, Schmitz N, Beeckman H, Koedam N. High-resolution time series of vessel density in Kenyan mangrove trees reveal a link with climate. New Phyt. 2005; 167: 425–435. 10.1111/j.1469-8137.2005.01415.x 15998396

[43] Rozendaal DMA, Zuidema PA. Dendroecology in the tropics: a review. Trees. 2011; 25: 3–16. 10.1007/s00468-010-0480-3

[44] Iftekhar MS and Saenger P. Vegetation dynamics in the Bangladesh Sundarbans mangroves: a review of forest inventories. Wetlands Ecol Manage. 2008; 16:291–312. 10.1007/s11273-007-9063-5

[45] Polidoro BA, Carpenter KE, Collins L, Duke NC, Ellison AM, Ellison JC, et al The loss of species: Mangrove extinction risk and geographic areas of global concern. PLoS One. 2010; 5(4). 10.1371/journal.pone.0010095 PMC2851656 20386710

[46] IPCC. Climate Change 2007: The physical science basis In: Solomon S, Qin D, Manning M, Chen Z, Marquis M, Averyt KB, et al, editors. Contribution of working group I to the fourth assessment report of the intergovernmental panel on climate change. Cambridge: Cambridge University Press; 2007.

[47] Ellison AM, Mukherjee BB, Karim A. Testing patterns of zonation in mangroves: scale dependence and environmental correlates in the Sundarbans of Bangladesh. J Ecol. 2000; 88: 813–824. 10.1046/j.1365-2745.2000.00500.x

[48] Mirza MMQ. Diversion of the Ganges water at Farakka and its effects on salinity in Bangladesh. Environ Manage. 1998; 22: 711–722. 9680539 10.1007/s002679900141

[49] Day PR. Particle fractionation and particle-size analysis In: Black CA, editor. Methods of soil analysis, Part 1, Agron Mongr. Madison: ASA; 1965 pp. 545–567.

[50] Shahid SA. Developments in salinity assessment, modeling, mapping, and monitoring from regional to submicroscopic scales In: Shahid SA, Abdelfattah MA, Taha FK, editors. Developments in soil salinity assessment and reclamation-innovative thinking and use of marginal soil and water resources in irrigated agriculture. Dordrecht, Heidelberg, New York, London: Springer; 2013 pp. 3–43.

[51] Tomlinson PB. The Botany of Mangroves. Cambridge: Cambridge University Press; 1994.

[52] Wils T, Robertson I, Eshetu Z, Touchan R, Sass-Klaassen U, Koprowski M. Crossdating Juniperus procera from North Gondar, Ethiopia. Trees. 2011; 25: 71–82. 10.1007/s00468-010-0475-0

[53] Schindelin J, Arganda-Carreras I, Frise E, Kaynig V, Longair M, Pietzsch T, et al Fiji: An open-source platform for biological image analysis. Nat Metho. 2012; 9: 676–682. 10.1038/nmeth.2019 PMC3855844 22743772

[54] Douglass AE. Crossdating in dendrochronology. J For. 1941; 39: 825–831.

[55] Baillie MGL, Pilcher JR. A simple program for tree-ring research. Tree-Ring Bull. 1973;33: 7–14.

[56] Eckstein D, Bauch J. Beitrag zur Rationalisierung eines dendrochronologischen Verfahrens und zur Analyse seiner Aussagesicherheit. Forstwissenschaftliches Centralblatt. 1969; 88, 230–250.

[57] 7. Rinn F. TSAP-WinTM user reference. Heidelberg: Rinn tech; 2003.

[58] Trouet V, Esper J, Beeckman H. Climate/growth relationships of Brachystegia spiciformis from the miombo woodland in South Central Africa. Dendrochronologia. 2010; 28: 161–171. 10.1016/j.dendro.2009.10.002

[59] Ridder De, den Bulcke JV, Acker JV, Beeckman H. Tree-ring analysis of an African long-lived pioneer species as a tool for sustainable forest management. For Ecol Manage. 2013b; 304: 417–426. 10.1016/j.foreco.2013.05.007

[60] Cook ER, Kairiukstis LA. Methods of dendrochronology: applications in the environmental sciences. Dordrecht: Kluwer Academic Publishers; 1990.

[61] Fritts HC. Tree rings and climate. London: Academic Press; 1976.

[62] Briffa KR, Jones PD. Basic chronology statistics and assessment In: Cook ER, Kairiukstis LA, editors. Methods of Dendrochronology: Applications in the environmental sciences. Dordrecht: Kluwer Academic Publishers; 1990 pp. 137–152.

[63] Biondi F, Waikul K. DENDROCLIM 2002: A C++ program for statistical calibration of climate signals in tree-ring chronologies. Comp Geosci. 2004; 30: 303–311. 10.1016/j.cageo.2003.11.004

[64] Van Oldenborgh GJ, Burgers G. Searching for decadal variations in ENSO precipitation teleconnections. Geophys Res Lett. 2005; 32 10.1029/2005GL023110

[65] Trouet V, Van Oldenborgh GJ. KNMI Climate Explorer: A web-based research tool for high-resolution paleoclimatology. Tree-Ring Res. 2013; 69: 3–13.

[66] Kennedy JJ, Rayner NA, Smith RO, Saunby M, Parker DE. Reassessing biases and other uncertainties in sea-surface temperature observations since 1850 part 1: measurement and sampling errors. J Geophys Res. 2011a; 11 6

[67] Kennedy JJ, Rayner NA, Smith RO, Saunby M, Parker DE. Reassessing biases and other uncertainties in sea-surface temperature observations since 1850 part 2: biases and homogenisation. J Geophys Res. 2011b; 116 10.1029/2010JD015220

[68] Trenberth KE, Stepaniak DP. Indices of El Niño evolution. J Clim. 2001; 14: 1697–1701. 10.1175/1520-0442(2001)014<1697:LIOENO>2.0.CO;2

[69] Mitchell TD, Jones PD. An improved method of constructing a database of monthly climate observations and associated high resolution grids. Int J Climatol. 2005; 25:693–712. 10.1002/joc.1181

[70] Chowdhury MR. The El Niño-Southern Oscillation (ENSO) and seasonal flooding- Bangladesh. Theor Appl Climatol. 2003; 76: 105–124. 10.1007/s00704-003-0001-z

[71] Ahasan MN, Chowdhary MAM, Quadir DA. Variability and trends of summer monsoon rainfall over Bangladesh. J Hydro Meteo. 2010; 7 10.3126/jhm.v7i1.5612

[72] Wang B, Fan Z. Choice of South Asian summer monsoon indices. Bull Amer Meteor Soc. 1999; 80: 629–638. 10.1175/1520-0477(1999)080<0629:COSASM>2.0.CO;2

[73] Mbow C. Chhin S, Sambou B, Skole D. Potential of dendrochronology to assess annual rates of biomass productivity in savanna trees of West Africa. Dendrochronologia. 2013; 31: 41–51. 10.1016/j.dendro.2012.06.001

[74] Worbes M. How to measure growth dynamics in tropical trees—A review. IAWA J. 1995; 16: 337–351.

[75] Trouet V, Mukelabai M, Verheyden A, Beeckman H. Cambial Growth Season of Brevi-Deciduous *Brachystegia spiciformis* Trees from South Central Africa Restricted to Less than Four Months. PLoS ONE. 2012; 7(10): e47364 10.1371/journal.pone.0047364 23071794 PMC3468463

[76] Schmitz N, Verheyden A, Kairo JG, Beeckman H, Koedam N. Successive cambia development in Avicennia marina (Forssk.) Vierh. is not climatically driven in the seasonal climate at Gazi Bay, Kenya. Dendrochronologia. 2007; 25:87–96. 10.1016/j.dendro.2006.08.001

[77] Devoe NN, Cole TG. Growth and yield in mangrove forests of the Federated States of Micronesia. Forest Ecol Manage. 1998; 103: 33–48. 10.1016/S0378-1127(97)00176-X

[78] Santini NS, Hua Q, Schmitz N, Lovelock CE. Radiocarbon dating and wood density chronologies of mangrove trees in Arid Western Australia. PLoS One. 2013; 8(11). 10.1371/journal.pone.0080116 PMC3827189 24265797

[79] Krauss KW, Doyle TW, Twilley RR, Rivera-monroy VH, Sullivan JK. Evaluating the relative contributions of hydroperiod and soil fertility on growth of south Florida mangroves. Hydrobiologia. 2006; 569: 311–324. 10.1007/s10750-006-0139-7

[80] Mwangi Theuri M, Kinyamario JI, Van Speybroeck D. Photosynthesis and related physiological processes in two mangrove species, Rhizophora mucronata and Ceriops tagal, at Gazi Bay, Kenya. African J Ecol. 1999; 37: 180–193. 10.1046/j.1365-2028.1999.00167.x

[81] Gopal B, Chauhan M. Biodiversity and its conservation in the Sundarban mangrove ecosystem. Aquat Sci. 2005; 68: 338–354. 10.1007/s00027-006-0868-8

[82] Sarker SK, Reeve R, Thompson J, Paul NK, Matthiopoulos J. Are we failing to protect threatened mangroves in the Sundarbans world heritage ecosystem? Sci Rep 2016; 6, 21234 10.1038/srep21234 26878801 PMC4754640

[83] Proseus TE, Boyer JS. Turgor pressure moves polysaccharides into growing cell walls of *Chara corallina*. Ann Bot. 2005; 95: 967–979. 10.1093/aob/mci113 15760911 PMC4246760

[84] Misra V, Pantina P, Chan SC, DiNapoli S. A comparative study of the Indian summer monsoon hydroclimate and its variations in three reanalyses. Clim Dyn. 2012; 39: 1149–1168. 10.1007/s00382-012-1319-y

[85] Kripalani RH, Inamdar S, Sontakke A. Rainfall variability over Bangladesh and Nepal: Comparison and connections with features over India. Int J Clim. 1996; 16: 689–703.

