# Supplementary figures and images for: Climatic Signals in Tree Rings of Heritiera fomes Buch.-Ham. in the Sundarbans, Bangladesh
Source: PLoS One. 2016 Feb 29;11(2):e0149788. doi: 10.1371/journal.pone.0149788 (PMC4771160; doi:10.1371/journal.pone.0149788)

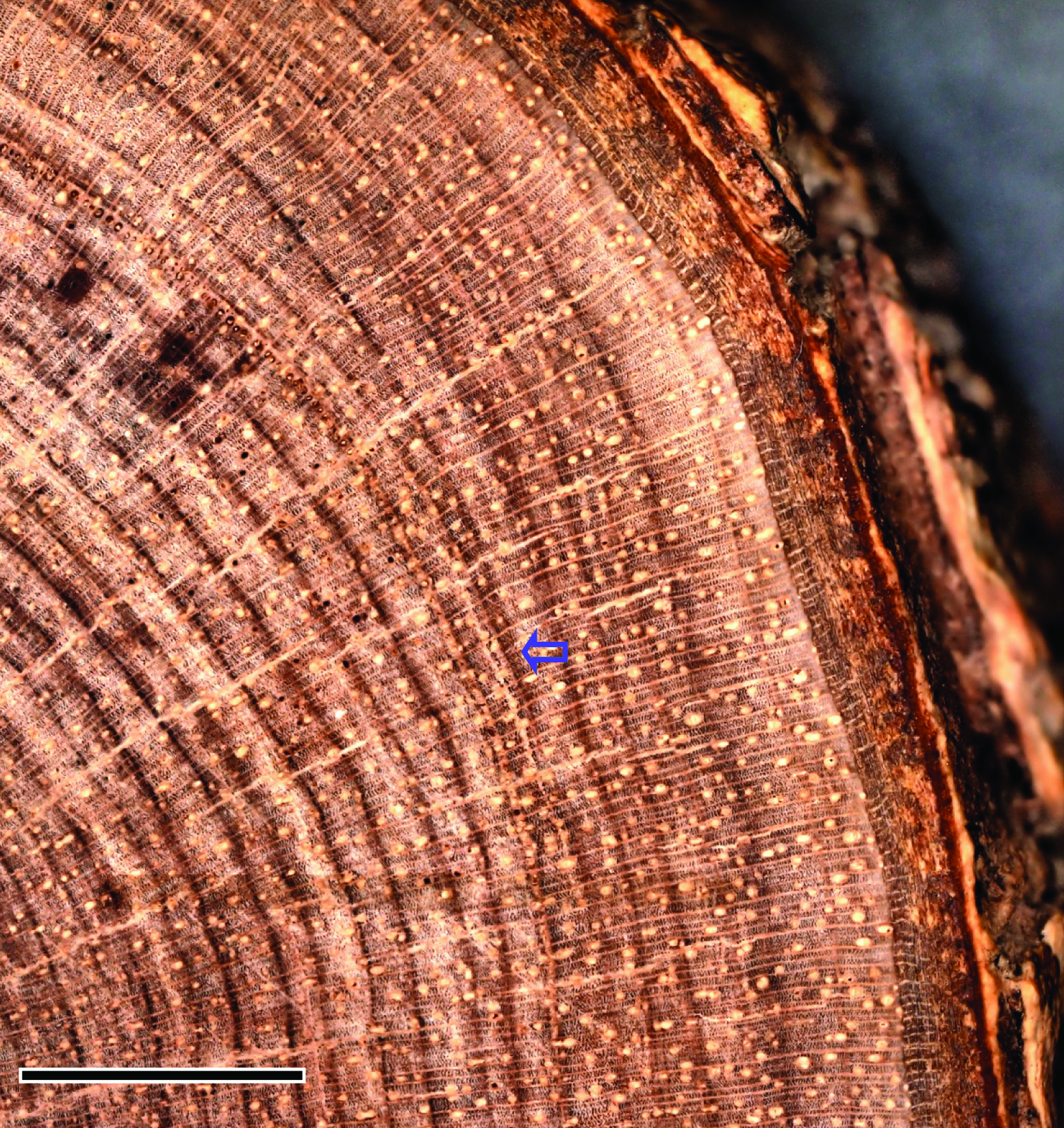

Supplement: S1 Fig — Scale bar = 5 mm. (TIF) [file pone.0149788.s001.tif]

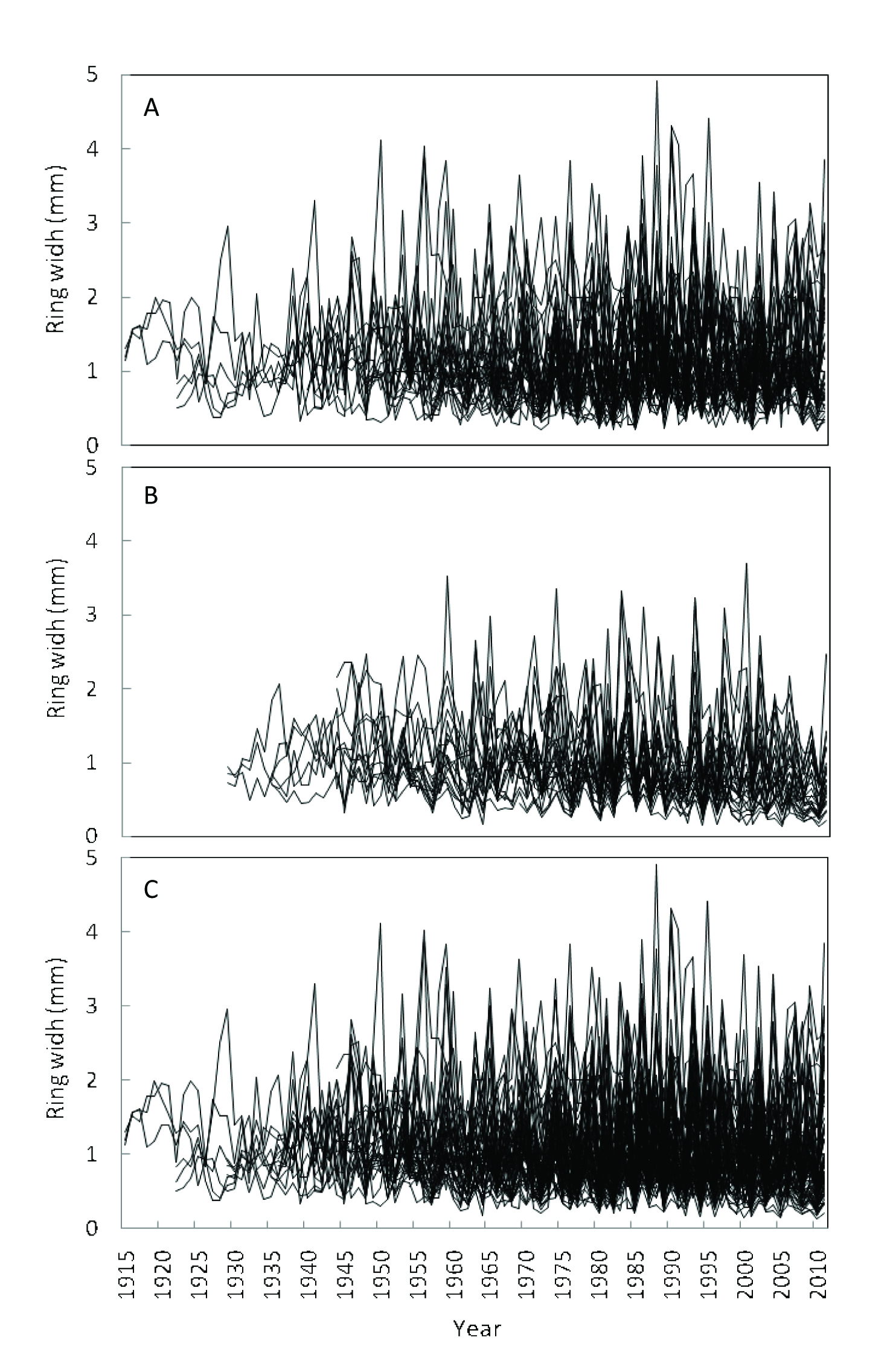

Supplement: S2 Fig — (TIF) [file pone.0149788.s002.tif]
